# Occupational Injury Patterns of Turkey

**DOI:** 10.1186/1749-7922-8-57

**Published:** 2013-12-28

**Authors:** Kaan Celik, Fevzi Yilmaz, Cemil Kavalci, Miray Ozlem, Ali Demir, Tamer Durdu, Bedriye Müge Sonmez, Muhittin Serkan Yilmaz, Muhammed Evvah Karakilic, Engin Deniz Arslan, Cihat Yel

**Affiliations:** 1 Numune Training and Research Hospital, Emergency department, Ankara, Turkey; 2 Baskent University Faculty of Medicine, Emergency department, Ankara, Turkey

**Keywords:** Emergency department, Occupational accident, Work, Cost

## Abstract

**Introduction and aim:**

Each year, a significant number of people die or become handicapped due to preventable occupational accidents or occupational diseases. The aim of this study was to investigate socio-demographic features, mechanism, causes, injury area, and sectoral features of occupational accidents in patients presented to our department.

**Materials and methods:**

The study was carried out retrospectively after local ethics committee approval. Age and sex of the patients, mechanism of injury, type and exact location of injuries were all evaluated. The groups were compared using Chi-Square test, Student’s T test and Kruskall-Wallis test. p value <0.05 was accepted as statistically significant.

**Results:**

Totally 654 patients were included in the study. 93.4% of patients were male, and mean age was 32.96 ± 5.97 (18–73) years. Sectoral distribution of accidents was statistically significant and mostly occurred in industrial and construction workers (p < 0.05, respectively). There is a statistically significant relationship between educational level and sector of the worker (p < 0.05). While the most frequent cause of admission to emergency department was penetrating injuries (36.4%), the least was due to multiple traumas (0.5%). Distribution of occupational accidents according to injury type was statistically significant (p < 0.05). The mean Injury Severity Score (ISS) was 9.79 ± 8.1. The mean cost of occupational injury was $1729.57 ± 8178.3. There was statistically significant difference between the sectors with respect to cost. Seventy-one patients (10.9%) recovered with permanent sequel and two (0.3%) died in hospital.

**Conclusion:**

Occupational accidents are most commonly seen in young males, especially in primary school graduated workers, and during daytime period.

## Introduction

World Health Organization (WHO) has defined occupational accident as “an unplanned event commonly leading to personal injury, damage to machinery and working equipment, and temporary halt of production” [1]. 270 million occupational injuries occur each year throughout the world, resulting 1.1 million deaths [2]. A considerable high number of people die or become handicapped each year due to preventable occupational accidents or occupational diseases [3-5].

Ankara is the second largest city of Turkey and has a population of 4.890.000 million. There are 10 organized industrial zone and since December 31, 2011 a total of 1,843 industrial companies have been registered in Ankara Chamber of Industry and a total of 286,860 workers have been employed in their establishments [6]. Small and Medium Industrial Enterprises (SMEs) account for the majority of industry in Ankara, Ankara is the 3rd largest industrialized province in Turkey (7% of total industrial enterprises) and today, 40% of industrial establishments in the area of production are machinery and metal industries [6]. According to the Health and Safety Executive Statistics 2011/12 of European Agency for Safety and Health, 173 workers were killed at work, a rate of 0.6 fatalities per 100,000 workers and 111,164 other injuries to employees were reported in United Kingdom [7]. Looking at the 2011 statistics of the Ministry of Labor and Social Security of Turkey, totally 62,903 occupational accidents were occurred and 2715 of these were in Ankara [8]. Due to proximity of our hospital to industrial zones, occupational accidents occurring in these areas are primarily admitted to our emergency department.

We aimed to investigate the socio-demographic features, mechanism, causes, and site of injury, and sectoral features in occupational accidents in patients presenting to Ankara Numune Training and Research Hospital emergency department.

## Materials and methods

This study enrolled 654 patients over the age of 18 years and admitted to Ankara Numune Training and Research Hospital emergency department with occupational accident between the dates 1 January 2011 and 31 December 2011. Patient files in hospital records system, patient assessment forms and judicial case reports prepared in emergency department were evaluated retrospectively after obtaining local ethics committee approval. Age and sex of the patients, mechanism of injury, cause of emergency department admissions, educational level and sector of worker, month of injury, hour of accident during the day, length of working hours, social security status, injured organ, state of preventive measures, disabled workers, injury severity score (ISS), hospital cost of occupational injuries, and site and healing status of injury were examined.

Collected data were analyzed using SPSS 19.0 software package programme. Normal distribution of descriptive statistical data was analyzed with Kolmogorov Smirnov test. The groups were compared using Chi-Square test, Student’s t test or Kruskall-Wallis test. The results were evaluated in a confidence interval of 95% and at a significance level of p < 0.05.

## Results

**Figure 1:**
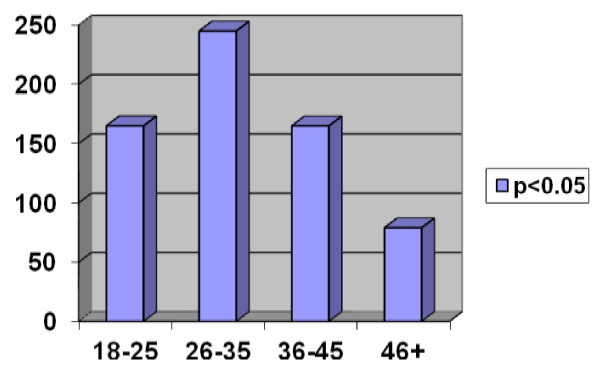
Distribution of cases by age range.

Among 654 patients admitted to Ankara Numune Training and Research Hospital due to occupational injury, 611 (93.4%) were male. Mean age of male and female patients were 32.9 ± 9.7 and 32.8 ± 9 years, respectively. There was no significant difference between both sexes with respect to age (p > 0.05) (Table 1). The number of occupational accidents increased in 26–35 age groups (37%). There was a significant difference between age groups with respect to occupational accident rate (p < 0.05) (Figure 1).

**Table 1:** Demographic characteristics according to gender

**Variable**		**Gender**				**p value**
		**Male**		**Female**		
		**n**	**%**	**n**	**%**	
Age (mean ± year)		611	32.9 ± 9.7	43	32.8 ± 9	0.934
Working experience (years)	0-1	131	96.3	5	3.7	
	1-5	297	92.2	25	7.8	
	5-10	79	91.9	7	8.1	0.366
	10+	104	94.5	6	5.5	
Mechanism	Machine Induced Hand Trauma	60	93.8	4	6.2	
	Glass Cut	43	89.6	5	10.4	
	Penetrating or Sharp Object Trauma	112	99.1	1	0.9	
	Blunt Object Trauma	150	94.9	8	5.1	0.04
	Foreign Object	11	100	0	0	
	Squeezing	35	100	0	0	
	Falls	139	89	17	11	
	Burns	44	91.7	4	8.3	
	Electric Injury	13	86.7	2	13.3	
	İntoxication	4	93.6	2	6.6	
Trauma region	Head & Neck	59	95.2	3	4.8	
	Face	25	100	0	0	
	Thorax	5	83.3	1	16.7	
	Abdomen	1	100	0	0	
	Pelvis	3	75	1	25	0.141
	Arm-Shoulder	70	93.3	5	6.7	
	Hand-Finger	264	95.7	12	4.3	
	Lower Extremity		90		10	
	Skin	22	84.6	4	15.4	
	Back-Vertebrae	27	87.1	4	12.9	

**Figure 2:**
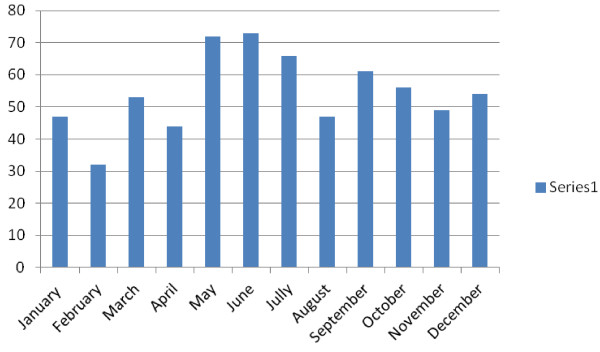
Monthly distribution of occupational accidents.

Monthly distribution of occupational accidents demonstrated that these accidents mostly occurred in May (12%) and least in February (4.9%). This distribution of occupational accidents was statistically significant (p < 0.05) (Figure 2).

The most occupational injury occurred in construction sector (28.7%). Sectoral distribution of accidents was statistically significant (p < 0.05) (Table 2). Analysis of occupational accidents with respect to educational level revealed that 251 (38.4%) were primary school graduate, 249 (38.1%) were high school graduate (Table 2).

**Table 2:** Relationship between sectoral distribution and education level

	**Education**				** p value**
**Sector (n)**	**İlliterate**	**Primary-Secondary school**	**High school**	**College**	
Industry	35	75	60	0	p < 0.001
Manufacturing	11	16	36	4	p < 0.001
Building	45	88	54	1	p < 0.001
Food	18	27	29	1	p < 0.001
Service	6	8	23	11	p < 0.001
Agriculture	2	1	1	0	p < 0.05
Transportation	5	5	15	0	p < 0.001
Woodwork	9	25	15	0	p < 0.001
Electricity	0	1	10	1	p < 0.001
Other	3	5	6	2	p < 0.001
Total	134	251	249	20	p < 0.001

Median working duration was 5.97 years (1 days-42 years). Most patients had a working duration of 1–5 years. Distribution of occupational accidents by working duration was statistically significant (p < 0.05). No significant difference was detected between male and female patients with respect to working duration (p > 0.05) (Table 1).

**Figure 3:**
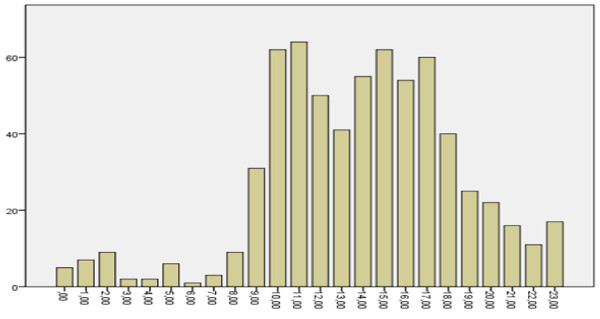
Hourly distribution of occupational accidents.

Time intervals of occupational accidents were as follows: 24^00^-08^00^ in 44 (6.7%) patients, 08^00^-16^00^ in 419 (64.1%) patients. The hourly distribution of occupational accidents was statistically significant (p < 0.05) (Figure 3).

The most common cause of admissions was cuts (36.4%). The distribution of occupational accidents by injury type was statistically significant (p < 0.05) (Table 3).

**Table 3:** The distribution of occupational accidents by inury type

**Injury type**	**Frequency (n)**	**(%)**
**Cuts**	238	36.4
**Soft tissue trauma**	152	23.2
**Amputation**	51	7.8
**Crush**	66	10.1
**Fracture-Dislocation**	77	11.8
**Burns**	48	7.3
**Electric Injury**	10	1.5
**Intoxication**	1	0.2
**Ocular Injury**	8	1.2
**Multiorgan Injury**	3	0.5
**Total**	654	100

The most frequent mechanism of occupational accidents was blunt object traumas in 158 (24.2%) cases. Distribution of patients according to mechanism of injury was given on Table 3. The mean ISS was 9.79 ± 8.1. Distribution of ISS score according to sector is summarized on Table 4.

**Table 4:** Distrubition of ISS score and cost according to sector

**Sector (n)**	**Cost (mean ± SD) ($)**	**p value**	**ISS**	**p value**
Industry	1427.5 ± 3443	p < 0.01	11.83 ± 9.2	p < 0.001
Manufacturing	732.16 ± 1657.2		8.26 ± 6.1	
Building	2836.44 ± 14039.7		9.17 ± 8	
Food	1547.68 ± 6055.3		7.82 ± 6.3	
Service	739.3 ± 2184.7		7.22 ± 5.3	
Agriculture	870.5 ± 651.6		15.75 ± 10.8	
Transportation	2077.32 ± 5997.2		9.2 ± 8.3	
Woodwork	1458.06 ± 2677.8		10.51 ± 6.7	
Electricity	1523.08 ± 2805.5		17.25 ± 15.3	
Other	591.37 ± 574.1		10.18 ± 6.9	
Total	1729.57 ± 8178.3		9.79 ± 8.1	

The most commonly affected body parts were upper extremities (53.7%, n = 351). Second most common region involved was lower extremities (15.3%, n = 100). Other data regarding affected body parts by occupational accidents are given on Table 1. No statistically significant difference was detected between males and females with respect to trauma region (p > 0.05).

The mean cost of occupational injury was $1729.57 ± 8178.3. Distribution of hospital cost according to sector was summarized on Table 4.

Of the patients, 549 (83.9%) were discharged after emergency department evaluation and treatment, while 105 (16.1%) patients were hospitalized. Two patients died at the admission ward. While 581 (88.8%) patients recovered without a sequel, 71 (10.9%) with sequel.

## Discussion

According to Social Security Institution statistics, the number of deaths due to occupational accidents in recent years are as follows: 1043 deaths in 80,602 occupational accidents in 2007, 865 deaths in 72,693 occupational accidents in 2008, 1171 deaths in 64,316 occupational accidents in 2009, and 1444 deaths in 62,903 occupational accidents in 2010. Moreover, hundreds of people have become handicapped each year [9]. These data indicate the importance of occupational accidents.

It has been reported that occupational accidents are more common in males (84-86%) [10-13], and our results correlate with the literature. More participation of males in work life possibly contributes to this finding. It has also been reported that occupational accidents are more common in 25–34 age group [9,10,12,14]. Majority of our study population were also in that age group. This may have been resulted from the fact that people from this age group belong to the productive population segment, and at the same time they are employed in more risky and hard jobs.

Karakurt et al. [15] reported that most occupational accidents occurred in December whereas Dizdar et al. [3] and Satar et al. [16] reported that occupational accidents increased in June, July and August. We observed that occupational accidents increased in May, June and July possibly because of air warming with resulting increase in volume of construction and agriculture sectors, with a parallel increase in manufacture of goods.

Previous studies showed that occupational accidents mostly occur with workers having less than 10 working years [12,17]. We found that rate of occupational accidents was the highest in workers with working years between 1–5 years, possibly because beginner workers are more careful at the beginning due to fear of making mistakes, but they may be progressively more careless as they gain experience.

Sayhan et al. [12] reported that occupational accidents occur mostly between 08.00-16.00 hours. Serinken et al. reported that the highest frequency of occupational accidents was observed between 08.00-12.00 hours [18]. We also found that most occupational accidents (64.1%) were seen between 08.00-16.00 hours. The frequency of occupational accidents increased during the day, gradually decreased at evening, and became minimized at night, possibly because only those working in night shifts remain at work at those hours. Another reason for the tendency of occupational accidents to occur more frequently during the first hours of a workday may be the fact that the workers begin to work without enough focus or adaptation to working environment.

Ozkan et al. [2] reported that majority of victims of occupational accidents worked in manufacturing and construction sectors (60%, 24%). In our study, 28.7% of the occupational accident cases worked in construction sector, 10.2% in manufacturing sector. Regional differences also brought about sectoral variations.

Serinken et al. [18] reported that cuts and lacerations had the highest rate with 40.1% followed by fractures-dislocations with a rate of 25.8%. In the study by Ozkan et al. [2], on the other hand, soft tissue injuries ranked first with a rate of 36.7% followed by cuts and fractures-dislocations with rates of 26.3% and 11.2%, respectively. Statistical data from Social Security Institution show that accidents related with sharp or penetrating objects ranked first with a rate of 13.3%, followed by falling from a height with 11.7% and machinery-related accidents with a rate of 10.6% [9]. We also detected that cuts had the highest rate of 36.4% followed by soft tissue trauma. The reason of a higher rate of cuts and soft tissue traumas may be increased safety level of the newly introduced machinery devices, an advanced level of alertness of workers while performing tasks that have a potential to cause a severe trauma, or carelessness of workers while performing tasks that have a potential to cause small traumas.

Ozkan et al. [2] reported that injuries due to penetrating objects/machinery had the highest rate (48.5%) followed by blunt object traumas (21.5%) and falls (18.9%). Jackson et al. [19] found that 54% of cases were due to penetrating objects/machinery. Our data indicate that 39.8% of the cases were due to penetrating objects/machinery followed by blunt object traumas (24.2%) and falls (23.9%). The primary reason responsible for the differences among these studies is the principle sector in the region of study. Some trauma mechanisms may be lower in women as a result of a negligible ratio of female workers to males in some sectors, as in the case of transportation and construction sectors. Thus, there may have been a significant difference between the trauma mechanism and sex.

Anders et al. reported a mean ISS of 19.2 for patients having a work accident [20]. Our patients had a mean ISS of 9.79 ± 8.1. We suggest that our patients had a low ISS since they sustained accidents of very low energy levels.

Anders et al. reported a mean hospital cost of €35.661 [20] while Asfaw et al. gave a figure of $2,328 [21]. Our patients had a mean cost of occupational injury of $1729.57 ± 8178.3. These costs don't include the money spent for rehabilitation. If labor force loss and rehabilitation expenses are added, the cost exceeds millions of dollars. We believe that the hospital cost was lower in our study as a result of our patients’ lower ISS score and cross-national differences of prices. Highest costs were observed in accidents of agriculture and transportation sectors. We think that accidents and costs can be reduced if universal safety measures are followed in construction sector and traffic rules observed in transportation sector.

It has previously been reported that the rate of occupational accidents increases when the educational level decreases [2,12]. Our results are consistent with the literature. Possible reasons of decreased occupational accident rate with increased educational level include the following: Educated persons may do their jobs more seriously; and they may take care of warning signs more compared to less educated people. In addition, educated persons may work in administrative positions, potentially avoiding dangerous jobs.

In our study, examination of injured body parts revealed that upper extremity injuries were at the top point with a rate of 53.7%. They were followed by, in descending order, lower extremity injuries (15.9%) and head-neck injuries (9.5%). Previous studies from our country have also revealed similar results [2-4]. Upper extremity injuries were the most common injuries since hands are intensely used at work.

It has been reported that 62-90% of patients admitting with occupational accident are discharged after first medical care at emergency departments [2,3,15,18]. In this study, 83.9% of cases were discharged after first medical care at emergency department, and 16.1% were hospitalized. No patients were referred to another healthcare facility as our center is a tertiary care center with all trauma-related surgical branches and a burn center readily available.

### Limitation of the study

A major limitations of the study was a retrospectiveness of it.

## Conclusion

Occupational accidents most commonly occur in young male workers, during daytime and primary school graduates.

## Competing interests

The authors declare that they have no competing interests.

## Author contributions

KC: conception and design, or acquisition of data, or analysis and interpretation of data, have given final approval of the version to be published. FY, MO, MEK: acquisition of data, MMS: revising it critically for important intellectual content; CK: analysis and interpretation of data or revising it critically for important intellectual content; AD, TD, EDA: have made substantial contributions to conception and design. MSY: have made substantial contributions to conception and design. All authors read and approved the final manuscript.
